# Arachidonic acid metabolites in pathogenic yeasts

**DOI:** 10.1186/1476-511X-11-100

**Published:** 2012-08-08

**Authors:** Ruan Ells, Johan LF Kock, Jacobus Albertyn, Carolina H Pohl

**Affiliations:** 1 Department of Microbial, biochemical and food Biotechnology, University of the Free State, Bloemfontein, 9300, South Africa; 2 Department of Microbial, biochemical and food Biotechnology, University of the Free State, PO Box 339, Bloemfontein, 9300, South Africa

**Keywords:** Arachidonic acid, Oxylipins, Pathogenic, Yeasts

## Abstract

Although most of what is known about the biology and function of arachidonic acid metabolites comes from the study of mammalian biology, these compounds can also be produced by lower eukaryotes, including yeasts and other fungi. It is also in this group of organisms that the least is known about the metabolic pathways leading to the production of these compounds as well as the functions of these compounds in the biology of fungi and yeasts. This review will deal with the discovery of oxylipins from polyunsaturated fatty acids, and more specifically the arachidonic acid derived eicosanoids, such as 3-hydroxy eicosatetraenoic acid, prostaglandin F_2α_ and prostaglandin E_2_, in yeasts starting in the early 1990s. This review will also focus on what is known about the metabolic pathways and/or proteins involved in the production of these compounds in pathogenic yeasts. The possible roles of these compounds in the biology, including the pathology, of these organisms will be discussed.

## Introduction

Fatty acids are the main components of lipids and play a key role as structural components of cellular membranes, affecting the physical state of the membranes, as storage lipids and as signaling molecules that impact the immune system in various ways [1]. Oxylipins is the collective term for oxygenated polyunsaturated fatty acids (PUFAs) and metabolites and includes the eicosanoids, which are an important group of oxygenated C20 PUFAs [2]. These compounds represent the prostaglandins, thromboxanes, prostacyclins, leukotrienes, lipoxins, hepoxilins, hydro(pero)xy fatty acids, hydroxylated fatty acids and epoxy derivatives [3,4]. In mammalian cells they are mainly synthesized from eicosatrienoic acid [20:3(n-6), dihomo-γ-linolenic acid (DGLA)], eicosatetraenoic acid [20:4(n-6), arachidonic acid (AA)] and eicosapentaenoic acid [20:5(n-3), EPA] [3] as well as from docosahexaenoic acid [22:6(n-3), DHA] [5]. They are synthesized through the actions of cyclooxygenases (COX) [6], lipoxygenases (LOX) [7], cytochrome P450s (CYP450s) [4,8,9], or non-enzymatic pathways [10]. However, in fungi the precursors for oxylipin production are usually octadecenoic acid [18:1(n-9), oleic acid], octadecadienoic acid [18:2(n-6), linoleic acid] and octadecatrienoic acid [18:3(n-3), linolenic acid] [11].

Most of what is known about oxylipins, such as eicosanoids, comes from the investigation of mammalian biology and very little is known about the biochemistry of eicosanoid production in the lower organisms, including yeasts. This review will focus on the occurrence of arachidonic acid derived oxylipins in pathogenic yeast, known metabolic pathways for the production of these oxylipins as well as the possible roles and significance of these compounds in the biology of pathogenic yeasts.

## Occurrence of eicosanoids in pathogenic yeasts

In the early 1990’s the presence of AA metabolites (including prostaglandins and 3-hydroxy (OH) fatty acids) in environmental yeasts belonging to the Dipodascaceae and Lipomycetaceae families was discovered [12-17]. Following this discovery, AA metabolites were also implicated in the pathogenesis of certain yeasts. Although, host cells produce eicosanoids, the pathogen can also contribute to this production. The production of a 3-OH fatty acid from exogenous AA was found in the pathogenic yeast, *Candida albicans *[18,19]. This compound was identified by GC-MS as 3,18-dihydroxy-5,8,11,14-eicosatetraenoic acid (3,18 di-HETE) and was associated with the hyphal forms, possibly playing a role in morphogenesis and pathogenicity. In biofilms of the closely related yeast, *C. dubliniensis*, the production of 3,18 di-HETE from exogenous AA was also found [20].

Noverr and co-workers [21] indicated by the use of ELISA assays that the pathogenic yeasts, *C. albicans *and *Cryptococcus neoformans,* have the ability to produce and secrete prostaglandins (PGs) *de novo* and that the addition of exogenous AA increased this production significantly. They referred to it as PGE_x_ due to the cross-reactivity observed with prostaglandins of the E class using prostaglandin immunoassays. Later, using mass spectrometry, it was verified as PGE_2 _[22,23]. *Candida albicans *and *Crypt. neoformans *can also produce other prostaglandins, including PGD_2 _and PGF_2α _as well as leukotrienes (LTB4, cysteinyl leukotrienes) from exogenous AA [24]. Similar results were obtained by Erb-Downward and co-workers [25] in *Crypt. neoformans*, however lysates from this yeast produced more PGF_2α_ compared to PGE_2_, in contrast to *C. albicans*, where PGE_2_ was the main prostaglandin produced. This eicosanoid production was found for planktonic cells, however the production of PGE_2_, sensitive to COX inhibitors, *de novo* by *C. albicans *biofilms has also been reported [26,27]. The COX inhibitors used in the latter study also inhibited biofilm formation. Interestingly, the addition of PGE_2_ together with acetylsalicylic acid (aspirin, ASA) completely removed biofilm inhibition by ASA. The authors concluded that biofilm development, morphogenesis and regulation of physiological processes in this yeast are regulated by COX-dependent synthesis of fungal prostaglandins.

During the last few years there has also been an increase in other non-*albicans Candida *species as opportunistic human pathogens [28]. These include *C. dubliniensis, C. glabrataC. krusei, C. tropicalis* and *C. parapsilosis*. Recently, it was found that *C. dubliniensis, C. glabrata* and *C. tropicalis* are also capable of producing PGE_2 _[29,30]. *Candida albicans, C. dubliniensis *and *C. tropicalis *produced considerable amounts of PGE_2_ whereas *C. glabrata* produced only trace amounts. Interestingly, in the presence of human keratinocytes, important in cutaneous immune responses, *C. albicans C. tropicalis* as well as *C. glabrata* produced 10-fold more PGE_2_ with the keratinocytes alone producing only trace amounts of PGE_2_. This indicates the involvement of PGE_2_ during host pathogen interactions, specifically during superficial infections.

The pathogenic dimorphic yeast, *Paracoccidioides brasiliensis*, also produced prostaglandins from endogenous and exogenous AA [31,32]. The use of COX inhibitors, indomethacin and piroxicam, not only decreased prostaglandin production but also viability of the yeast.

## Biosynthesis of yeast eicosanoids

The biosynthetic pathway for eicosanoid production in mammalian cells has been well studied and is used as a model to try and identify enzymes involved in this pathway in lower organisms, including fungi. The specific AA metabolites produced *in vivo* in mammalian cells are dependent upon the most active enzymes in specific tissues [33]. Cyclooxygenases, LOX, CYP450s and β–oxidation enzymes are known to add hydroxyl groups to AA [34].

Limited information is, however, available regarding the mechanisms involved in eicosanoid production in yeasts. Enzymatic involvement in these pathways was indicated by incubating AA together with boiled lysates of either *C. albicans *or *Crypt. neoformans *[22,23,25]. This lead to a significant reduction in PGE_2_ produced, suggesting the presence of a denaturable enzymatic pathway in these yeasts. Brodhun and Feussner [35] speculated about the unlikelihood of the existence of a specific prostaglandin pathway in fungi. They ascribed these reactions to be similar to a known isoprostane type of non-specific lipid peroxidation reaction that can be catalyzed by any protein harboring iron as cofactor.

The use of different enzyme inhibitors is widely applied in order to identify the pathways and putative enzymes involved in eicosanoid production by fungi. There are a number of studies that used COX inhibitors, including ASA and other non-steroidal anti-inflammatory drugs (NSAIDs), as well as LOX inhibitors, to identify possible mechanisms involved in fungal eicosanoid production [15,25,36,37], however this has not been able to provide conclusive evidence.

### *Candida albicans*

Initially Noverr and co-workers [21] speculated that COX-like enzymes had to be present in *C. albicans*. In their studies they used different COX inhibitors (i.e. etodolac, indomethacin and piroxicam), to evaluate prostaglandin production. They found that all these inhibitors not only decreased prostaglandin production but also decreased the viability of these cells. This suggests that the decrease was not due to a specific inhibition of the enzyme but rather due to an effect on cell viability or that prostaglandin production could regulate the viability of the yeast [22]. Later, using non-selective mammalian COX inhibitors, ASA, indomethacin and resveratrol, and the LOX inhibitor, nordihydroguaiaretic acid (NDGA) (also known as a COX inhibitor) the production of PGE_2_ was reduced without affecting viability [23]. Similar results were obtained for *C. albicans *and *C. dubliniensis* biofilms using ASA and NDGA [29]. However, the use of the selective COX-2 inhibitor, CAY10404 [3-(4-methylsulphonylphenyl)-4-phenyl-5-trifluoromethylisoxazole], had no effect on PGE_2_ production, suggesting that enzymes distinct from mammalian COX and LOX are responsible for PGE_2_ production in *C. albicans *[23]. This agrees with the BLAST results used to search the genomes of *C. albicans *for COX and LOX homologues, which did not reveal any sequences with significant homology to mammalian COX and LOX [38,39]. This was followed by the identification of two non-COX/LOX-related enzymes, involved in PGE_2_ production in *C. albicans *[23]. These enzymes were identified as a fatty acid desaturase, Ole2p, and a multicopper oxidase or laccase homologue, Fet3p. The importance of these enzymes was illustrated by indicating that mutants lacking the *OLE2* or *FET3* gene had a reduced production of PGE_2_. However, this did not completely inhibit PGE_2_ production, suggesting that other enzymes are also involved.

Recently, the involvement of multicopper oxidases in the production of PGE_2_ by both *C. albicans *and *C. dubliniensis* biofilms, as well as the possible role of CYP450s in prostaglandin production, possibly upstream of the multicopper oxidases were also indicated [29]. This was done using different CYP450 inhibitors [6-(2-propargyloxyphenyl)hexanoic acid and 1-aminobenzotriazole] and multicopper oxidase inhibitors (ammonium tetrathiomolybdate and sodium azide). These inhibitors significantly decreased PGE_2_ production without affecting cell biomass and viability of *C. albicans *and *C. dubliniensis* biofilms. The availability of the genomes indicated the presence of 12 CYP450s in *C. albicans *and 10 CYP450s in *C. dubliniensis* compared to the only 3 in *S. cerevisiae *[40]. This may indicate the involvement of CYP450s in pathogenesis.

### *Cryptococcus neoformans*

Since the COX inhibitor, indomethacin, could reduce prostaglandin production in *Crypt. neoformans*, it was initially speculated that the enzyme involved was COX-related [21]. However, similar to *C. albicans*, the genome of *Crypt. neoformans * did not reveal any sequence homology to mammalian COX and LOX [22,25]. The study by Erb-Downward and Huffnagle [22] did not observe this inhibitory effect in the presence of the COX inhibitors, ASA and indomethacin, suggesting that other non-COX enzymes are involved. It must, however, be noted that the difference in especially incubation time used in the latter study, may have contributed to the observed difference in results.

In another study, Erb-Downward and co-workers [25] indicated that the polyphenolic LOX inhibitors, caffeic acid, NDGA and resveratrol inhibited both PGE_2_ and PGF_2α_ production in *Crypt. neoformans*, even though a LOX homologue is absent. This lead to the identification of a multicopper oxidase, laccase, known to bind polyphenols, as an enzyme involved in prostaglandin production in *Crypt. neoformans*. Laccase alone did not convert the PGE_2_ precursors (AA or PGH_2_) to PGG_2_ or new prostaglandins, but it did convert PGG_2_ to PGE_2_ and 15-keto-PGE_2_. This suggests that multicopper oxidases might play a significant role in eicosanoid production by this pathogenic yeast. However, it is not the only enzyme involved, and questions still need to be answered regarding the enzymes upstream of the multicopper oxidase. In addition, it has been speculated that enzymes belonging to the Old Yellow Enzyme family might be involved in this pathway, leading to the production of PGF_2α_ from PGE_2 _[25].

### *Paracoccidioides brasiliensis*

Similar to *C. albicans *and *Crypt. neoformans*, it is speculated that a COX pathway is involved in the production of prostaglandin by the dimorphic yeast, *P. brasiliensis *[31,32]. This was indicated by the use of indomethacin and piroxicam, which not only inhibited prostaglandin production but also fungal viability. The authors suggest a COX-dependent metabolic pathway is involved and that, similar to *C. albicans*, prostaglandins have a possible role in fungal survival.

## Biological activity of eicosanoids

The production of eicosanoids by mammalian cells is in response to mechanical factors or chemical stimuli, such as cytokines, or in response to pathogen invasion [41]. They act similar to hormones, as potent biological regulators and are involved in many systems such as the cardiovascular, renal, reproduction and the immune system [42,43]. The immunomodulatory properties of eicosanoids have been studied intensively in mammalian cells with a single eicosanoid capable of having pleiotropic functions [41,44]. This includes different physiological and pharmacological effects on different cell types. These effects are mainly due to the existence of multiple receptors for each lipid species on plasma membranes. Eicosanoids are known to function through G-protein-coupled receptors (GPCRs), known as guanine nucleotide regulatory proteins, to elicit their pharmacological and signaling profiles [3,44]. The activated trimeric G-proteins affect the concentrations of the second messengers, cyclic AMP (cAMP), or intracellular ions such as K^+^. This occurs through the stimulation or inhibition of adenylate cyclase or the opening or closing of K^+^ channels.

CD4^+^ T cells differentiate into CD4^+^ T helper (Th) cells, Th1 or Th2, in response to antigens or cytokines to eliminate pathogens [45]. These Th cells secrete different cytokines and have different functions during an immune response. The Th1 cytokines are involved in activating macrophages and cytotoxic T cells, known as cell-mediated/protective immunity against intracellular pathogens [46]. In addition, the Th2 cytokines are involved in humoral immunity by helping B cells to produce antibodies. However, an imbalance in Th1/Th2 responses can be detrimental to the host, leading to serious autoimmune diseases while these responses can also negatively regulate each other [47]. Interestingly, pathogen studies suggested that eicosanoid production, especially PGE_2_, could shift these Th responses in favor of the pathogen [47,48]. Therefore, the activation of Th1 and/or Th2 responses may correlate to the occurrence of resistance and susceptibility to infections.

Both host and pathogen are capable of producing PGE_2_ during an infection indicating that both can modulate immune responses [48,49]. These biological effects of prostaglandins on the immune system and the enhanced production of prostaglandins and leukotrienes by pathogenic yeasts may lead to the intracellular survival followed by chronic and disseminated infections [50]. This can be illustrated by the ability of these eicosanoids to down-regulate macrophage functions as mentioned above for mammalian eicosanoids.

*Candida* infections stimulate both the innate and adaptive immune responses [51-53]. Although several studies indicated that *C. albicans *and *Crypt. *stimulate the production of Th1-type cytokines leading to protective immunity or acquired resistance against these pathogens [53-57], the production of PGE_2_ during infections inhibits Th1 responses and has an inducing effect on Th2 and Th17 responses [49,58,59]. It is known that Th1 responses are critical for protection against candidiasis whereas Th2 responses are less important [60]. Additionally, Th2 responses are non-protective against pathogens and lead to chronic or disseminated infections [51]. Th17 responses play an important role in autoimmune diseases as well as the control of fungal infections by initiating and maintaining inflammation [61]. Additionally, the uncontrolled production of Th17 cytokines can be harmful to the host during systemic infections [59]. *Candida albicans *PGE_2_ enhances Th17 responses by stimulating the production of IL-17 and IL-22. Similarly, the monocyte subset, CD14^++^ CD16^-^, produce more PGE_2_ and also induces a greater Th17 response compared to the other monocyte subsets, CD14^+^ CD16^+^, in response to *C. albicans *[62]. The Th17 responses are induced by the presence of the pathogen associated molecular patterns, mannan and β-glucan from *C. albicans *[61,63]. So the production of PGE_2_ during infections might be beneficial for the pathogens. Another important factor induced by PGE_2_, is tissue eosinophilia, leading to tissue damage, which is a common feature of some chronic fungal infections [49].

The PGE_x_ (PGE_2_) produced by both *C. albicans *and *Crypt. neoformans *was found to be biologically active on both yeast and mammalian cells [21,64]. It had immunosuppressive effects in mammalian cells by down-modulating chemokine production, tumor necrosis factor alpha (TNFα) production and splenocyte proliferation while up-regulating IL-10 production (Th2 responses). The biological activity on yeast was indicated through the stimulation of germination in *C. albicans*, similar to synthetic PGE_2_ and thromboxane B_2_ (TXB_2_) [64]. Similar results were obtained by Kalo-Klein and Witkin [65] using commercial PGE_2_, suggesting that morphogenesis (yeast-to-hyphae transition) is induced in *C. albicans *by a PGE_2_ caused increase in cAMP levels. The increase in intracellular cAMP levels in guinea-pig tracheal epithelial cells due to commercial PGE_2_ was also observed [66]. However, other prostaglandins i.e. PGI_2_, PGF_2α_ and PGD_2_ did not exert any effect on cAMP. Interestingly, PGF_2α_ had no effect on germination of *C. albicans *[65]. This suggests that the other prostaglandins, PGI_2_ and PGD_2_, may also not affect germination by *C. albicans.* The PGE_2_ precursor, AA as well as other long chain fatty acids (i.e. C18 fatty acids) did not have any effect on germination [64].

Morphogenesis in pathogenic fungi is often associated with increased virulence and mucosal invasiveness [67] and the start of biofilm formation, with infections by *C. albicans *mainly caused by biofilms [68]. This suggests that prostaglandin production, especially PGE_2_, might be an important virulence factor. However, the exact role of prostaglandins in morphogenesis and biofilm development is complex and remains unclear. Although, Alem and Douglas [27] indicated the production of PGE_2_ by *C. albicans* biofilms, PGE_2_ did not function as a QS molecule, with no correlation found between PGE_2_ production and cell density. In addition, 3,18-diHETE was shown, through immunofluorescence microscopy using a polyclonal antibody, specific against chemically synthesized 3-OH fatty acids [69], to be mainly associated with the hyphal forms of *C. albicans* and not the yeast form [18]. This might play a role in the anchorage of these cells to host cells during infections. This indicates that 3-OH fatty acid production may be an important virulence factor in *C. albicans *as well as in *C. dubliniensis*, since 3,18-diHETE production has also been found in this closely related yeast [20]. Interestingly, the related 3-OH AA metabolite, 3*R*-HETE, produced by the non-pathogenic yeast *D. uninucleata*, was also reported to have pro-inflammatory actions in mammalian cells by affecting signal transduction processes in human neutrophils and tumor cells [70,71]. Ciccoli and co-workers [72] also indicated that 3-HETE can be used as a substrate for mammalian COX-2, activated by *C. albicans *in a HeLa infection model [73], to produce pro-inflammatory 3-OH PGE_2_. This metabolite increased inflammation in host cells. The immunomodulatory activities of this compound were illustrated through the increased upregulation of IL-6 gene expression (an enhanced Th2 response), in comparison to PGE_2_, as well as cAMP levels, similar to PGE_2_, in Jurkat T-cells and lung adenocarcinoma cells (A549 cells), respectively [49,72].

## Conclusions

This review indicates that AA metabolites are widely distributed in the fungal domain. The limited available knowledge about the possible pathways or enzymes involved in fungal eicosanoid production compared to mammalian cells, is evident. In addition, when COX and LOX inhibitors are used to identify the pathways involved, it should be noted that these inhibitors are non-selective, therefore they could inhibit various enzymes needed for fungal viability, thus leading to cell death. The so called non-prostaglandin mediated effects by different NSAIDs have been indicated before in mammalian cells [74]. These include the interactions of NSAIDs with biological membranes and influencing important cell functions such as transmembrane anion transport, oxidative phosphorylation, enzyme activity as well as the uptake of AA. These mechanisms, together with the effect of ASA on the mitochondrial activity of various yeasts [75], might provide a possible explanation for the observed effects in the different studies. Additionally, some of these inhibitors used, such as NDGA, also have antioxidative properties which could also lead to the observed decrease in prostaglandin production [76].

The statement that PGE_2_ is responsible or involved in fungal viability should be interpreted with care. Although, Alem and Douglas [26] as well as de Quadros and co-workers [77] indicated that NSAID mediated inhibition of biofilm formation by *C. albicans *can be overcome by the addition of PGE_2_, the exact role of PGE_2_ during *C. albicans *biofilm formation is still unclear and needs further investigation.

The importance of eicosanoids during host pathogen interactions was also emphasized. More specifically, the immune response, the system that is responsible for protecting us against pathogens, can be altered through the production of eicosanoids. The production of these lipid mediators functions as components of a complex chemical signaling system between host and pathogen. Additionally, the effects of prostaglandins and their fatty acid precursors might be species specific, but that it does play an important role during pathogenesis cannot be ignored.

## Competing interests

The authors declare that they have no competing interests.

## Authors’ contributions

CHP was responsible for the layout and initial idea for the manuscript. RE and CHP wrote the manuscript. JLFK and JA participated in researching the literature and critical discussion of the work. All authors read and approved the final manuscript.
